# Combined inhibition of Wee1 and Chk1 as a therapeutic strategy in multiple myeloma

**DOI:** 10.3389/fonc.2023.1271847

**Published:** 2023-12-06

**Authors:** Angélique Bruyer, Laure Dutrieux, Hugues de Boussac, Thibaut Martin, Djamila Chemlal, Nicolas Robert, Guilhem Requirand, Guillaume Cartron, Laure Vincent, Charles Herbaux, Malik Lutzmann, Caroline Bret, Philippe Pasero, Jérôme Moreaux, Sara Ovejero

**Affiliations:** ^1^ Diag2Tec, Montpellier, France; ^2^ Institute of Human Genetics, UMR CNRS-UM 9002, Montpellier, France; ^3^ Department of Biological Hematology, CHU Montpellier, Montpellier, France; ^4^ Department of Clinical Hematology, CHU Montpellier, Montpellier, France; ^5^ University of Montpellier, UFR Medicine, Montpellier, France; ^6^ Institut Universitaire de France (IUF), Paris, France

**Keywords:** multiple myeloma, Chk1, Wee1, therapeutic targets, replicative stress

## Abstract

Multiple myeloma (MM) is a hematological malignancy characterized by an abnormal clonal proliferation of malignant plasma cells. Despite the introduction of novel agents that have significantly improved clinical outcome, most patients relapse and develop drug resistance. MM is characterized by genomic instability and a high level of replicative stress. In response to replicative and DNA damage stress, MM cells activate various DNA damage signaling pathways. In this study, we reported that high *CHK1* and *WEE1* expression is associated with poor outcome in independent cohorts of MM patients treated with high dose melphalan chemotherapy or anti-CD38 immunotherapy. Combined targeting of Chk1 and Wee1 demonstrates synergistic toxicities on MM cells and was associated with higher DNA double-strand break induction, as evidenced by an increased percentage of γH2AX positive cells subsequently leading to apoptosis. The therapeutic interest of Chk1/Wee1 inhibitors’ combination was validated on primary MM cells of patients. The toxicity was specific of MM cells since normal bone marrow cells were not significantly affected. Using deconvolution approach, MM patients with high *CHK1* expression exhibited a significant lower percentage of NK cells whereas patients with high *WEE1* expression displayed a significant higher percentage of regulatory T cells in the bone marrow. These data emphasize that MM cell adaptation to replicative stress through Wee1 and Chk1 upregulation may decrease the activation of the cell-intrinsic innate immune response. Our study suggests that association of Chk1 and Wee1 inhibitors may represent a promising therapeutic approach in high-risk MM patients characterized by high *CHK1* and *WEE1* expression.

## Introduction

Multiple Myeloma (MM) is a plasma cell neoplasia arising from the malignant transformation of post follicular B cells. MM is characterized by extensive molecular and clinical heterogeneity (1, 2). The DNA damage response (DDR) coordinates the biological response to protect cells from genotoxic stress by sensing DNA damage and replication stress. These signal transduction pathways, among others, coordinate DNA repair, control cell cycle, and determine cell destiny. The genomic integrity of cancer cells is particularly challenged by DNA damage and replication stress, as well as by metabolic, mitotic, oxidative, and proteotoxic stressors, as a result of their dysregulated proliferation (3, 4). In MM, deregulation of DNA repair pathways may maintain and promote genetic instability and drug resistance to genotoxic agents by specific mechanisms that tolerate or rapidly bypass DNA damages to support MM cell survival and proliferation (3). Our group previously reported the significant role of BLM and RECQ1 DNA helicases in replication stress tolerance and drug resistance in MM (5–7). In response to replicative and DNA damage stress, MM cells activate various DNA damage signaling pathways that include the ATR, Chk1 and Wee1 kinases (3, 4, 6). Chk1 is activated by ATR after DNA damage or replicative stress and promotes cell cycle arrest through the regulation of S and G2 checkpoints. Wee1 is a kinase controlling G/M and S phase checkpoints via phosphorylation of the cyclin-dependent kinases CDK1 and CDK2. Furthermore, Wee1 inhibition prolongs mitosis in a range of cancer cells and makes them more susceptible to chemotherapy-induced mitotic catastrophe. It has been reported that the inhibition of Chk1, Chk2 or Wee1, as monotherapy or in combination with DNA damaging agents, induces apoptosis in several cancers, including hematological malignancies (8, 9).

Synthetic lethality refers to an interaction between two genes when the perturbation of either gene alone is viable but the simultaneous perturbation of both genes leads to cell death. Combination of Chk1 and Wee1 inhibitors demonstrated synergistic effects in different tumors including hematological cancers (8, 10). Chk1 inhibitors as well as Wee1 inhibitors have entered clinical trials in association with radiation or chemotherapy (11). In this manuscript, we identified that concomitant high expression of Chk1 and Wee1 delineate a subgroup of MM patients with a poor outcome in independent cohorts of MM patients and we intended to explore the therapeutic interest to combine Chk1 and Wee1 inhibitors as a therapeutic strategy in MM.

## Methods

### Human myeloma cell lines, genotoxic agents and inhibitors

XGs HMCLs cell lines have been derived in our laboratory as previously described (12) and are IL-6 dependent. XGs cell lines are routinely maintained in RPMI 1640 GlutaMAX medium (61870044, Gibco) supplemented with 10% fetal calf serum (CVFSVF00 01, Eurobio) and with IL-6 (2 ng/ml) (12). AMO-1, LP1 and OPM2 were purchased from DSMZ (Braunsweig, Germany) and RPMI8226 from ATCC (Rockville, MD, USA). These cell lines are maintained in RPMI 1640 GlutaMAX medium (61870044, Gibco) supplemented with 10% fetal calf serum (CVFSVF00 01, Eurobio). All the HMCLs were authenticated according to their short tandem repeat profiling. Affymetrix U133 plus 2.0 microarrays data have been deposited in the ArrayExpress public database under accession numbers E-TABM-937 and E-TABM-1088 (13).

Drugs used in this study: Chk1 inhibitor (AZD7762, Selleck Chemicals), Wee1 inhibitor (MK1775, Selleck Chemicals) and melphalan (Y0001457, European Pharmacopoeia Reference Standard).

### Gene expression analysis and gene set enrichment analysis

Normal bone marrow plasma cells (n=5) and patients’ MMCs (n=206) were purified using anti-CD138 MACS microbeads (Miltenyi Biotec, Bergisch Gladbach, Germany) and their gene expression profile (GEP) obtained using Affymetrix U133 plus 2.0 microarrays as described (Array Express public database (E-MTAB-372)) (14) or RNA sequencing as described (5, 15). Publicly available cohorts of newly-diagnosed MM patients treated with high dose melphalan and autologous hematopoietic stem cell transplantation (UAMS-TT2 and TT3 (GSE24080), and Hovon (GSE19784) cohorts) were also used. Gene expression data were normalized with the MAS5 algorithm and analyses processed with GenomicScape (http://www.genomicscape.com) (16). Gene Set Expression Analysis (GSEA) was used to identify genes and pathways differentially expressed between populations. Difference in overall survival between groups of patients was assayed with a log-rank test and survival curves plotted using the Kaplan–Meier method (Maxstat R package) (17).

### Analysis of Chk1 and Wee1 inhibitors’ toxicity on primary multiple myeloma cells

Bone marrow samples from untreated MM patients (n = 8) were obtained at the University Hospital of Montpellier after patients’ written informed consent in accordance with the Declaration of Helsinki and agreement of the Montpellier University Hospital Centre for Biological Resources (DC-2008-417). Bone marrow mononuclear cells are cultured with IL-6 (2ng/ml) (6) seeded at 5x10^5^cells/mL in RPMI 1640 medium, 5% FCS, 2ng/mL IL-6, and cultured with or without AZD7762 (39 nM, 156 nM, 625 nM or 2500 nM) for 4 days as described (6) in presence of IC20 of MK1775 (39 nM). In each culture group, viability and cell count were assayed and MM cell cytotoxicity was assessed by flow cytometry. MM plasma cells (CD138^+^) were detected using anti-CD138-phycoerythrin monoclonal antibody (Immunotech, Marseille, France) and all CD138^-^ cells were analyzed as non-myeloma cells as described (6).

### Proliferation assays and synergy analyses

For IC50 determination, HMCLs were seeded at 10000 cells/well and cultured for 4 days in 96-well flat-bottom plates in presence of increasing concentrations of AZD7762 or MK1775. Cell proliferation was evaluated using CellTiter-Glo (CTG) Luminescent Assay (G7573, Promega) according to the manufacturer’s protocol and luminescence was measured using a Centro LB 960 luminometer (Berthold Technologies, Bad Wildbad, Germany). IC50 for each HMCL was calculated using non-linear regression analysis in GraphPrism software (5).

For evaluation of AZD7762 and MK1775 synergy, increasing concentrations of AZD7762 were combined with IC20 of MK1775 (AMO1: 200nM; XG6: 300nM; XG7: 550nM). Cell growth was evaluated with CTG reagent as described above. Significant synergy have been calculated by the method of Chou and Talalay (18).

### Apoptosis analysis (annexin V quantification)

Cells were treated as indicated, and 10^5^ cells per condition were processed with the Annexin V kit (556421, BD Biosciences) according to the manufacturer’s instructions. Apoptotic cells (AnnexinV+) were quantified by flow cytometry.

### Cell cycle, DNA damage and apoptosis analysis by flow cytometry

Cells were culture in 12-wells plates for 4 days. For cell cycle analysis, BrdU (10 μg/ml) was added to the cultures for 1.5 h at the end of the treatments. Cells were collected and processed with the “Apoptosis, DNA damage and cell proliferation kit” (BD Pharmingen), following the manufacturer’s protocol. Samples were acquired with a Fortessa flow cytometer (BD biosciences) and results were analyzed with Kaluza software. Cells were assessed to the different phases of the cell cycle based on their DNA content (DAPI staining) and BrdU +/- staining. Cells with DNA damage were identified as “γH2AX positive” and apoptotic cells as “PARP cleavage positive” and expressed as percentage of the global population.

### Western blot analysis

Cells were treated as indicated and resuspended in Laemmli buffer 1X (400 μl/1 million cells), and boiled at 95°C for 5 min. 20 μl of lysate were loaded onto SDS-page acrylamide gels. Membranes containing proteins were incubated with primary antibodies diluted in 3% BSA-TBS-0.1% Tween overnight at 4°C, washed 3 times with TBS-0.1% Tween (10 min, RT), incubated with secondary antibodies anti-rabbit-HRP (7474, Cell Signaling) or anti-mouse-HRP (7076, Cell Signaling) (1:3000), washed 3 times, and developed with ECL (1705061, BioRad). Image acquisition was performed with BioRad system and processed with ImageLab software.

Unless otherwise stated, all primary antibodies used in this study were used at a 1:1000 dilution. Wee1 (sc-9037, Santa Cruz, 1:500), pSer345-Chk1 (2348S, Cell Signaling), Chk1 (ab40866, Abcam), pTyr15-Cdk1 (4539S, Cell Signaling), Cdk1 (9112S, Cell Signaling), Cdc25A (3652S, Cell Signaling), γH2AX (05-636, Millipore), pSer15-p53 (9284S, Cell Signaling), p53 (9282S, Cell Signaling), Caspase 3 (9662S, Cell Signaling), Caspase 8 (9746S, Cell Signaling), Caspase 9 (9502S, Cell Signaling), Tubulin (AB_1157911, DSHB).

### Immunofluorescence of γH2AX and 53BP1

Cells were treated as indicated, deposited on poly-lysine coated slides (J2800AMNZ, Thermo Scientific) using a Cytospin 4 centrifuge (Thermo Scientific) (600 rpm, 10 min, RT), fixed with 4% paraformaldehyde-PBS (10 min, RT), permeabilized with 0.1% Triton X-100-PBS (10 min, RT), blocked in 1% BSA-PBS (30 min, RT), incubated with primary antibodies (anti-γH2AX (05-636, Millipore, 1:200) and anti-53BP1 (NB100-04, 1:300)) diluted in saturation buffer overnight at 4°C. Then, slides were washed 3 times with 0.1% Tween-20-PBS (5 min, RT), and incubated with secondary antibodies (Goat anti-Rabbit AF647 (A21244, Thermo Scientific) and Goat anti-Mouse AF488 5A11029, Thermo Scientific)) (1:500) in saturation buffer (45 min, RT) protected from light, washed 3 times, and DNA was stained with DAPI (20 mg/ml) diluted in dH_2_O for 5 min. Slides were then rinsed 3 times with dH_2_O, air dried and mounted with ProlongGold (P36930, Invitrogen) and let to dry overnight. Images were acquired with a ZEISS Axio Imager Apotome microscope and analyzed with Omero server. Foci quantification was performed with Image J.

### Gene expression profiling of bone marrow microenvironment

Purified MM cells from patients and normal bone marrow environment fraction RNA sequencing was done as previously described (15, 17). The RNA sequencing (RNA-seq) library preparation was done with 150 ng of input RNA using the Illumina TruSeq Stranded mRNA Library Prep Kit. Paired-end RNA-seq were performed with Illumina NextSeq sequencing instrument (Helixio, Clermont-Ferrand, France). RNA-seq read pairs were mapped to the reference human GRCh37 genome using the STAR aligner. All statistical analyses were performed with the statistics software R (version 3.6.2; https://www.rproject.org), and R packages developed by Bioconductor project (https://www.bioconductor.org) (19). The expression level of each gene was summarized and normalized using the DESseq R/Bioconductor package (20). CIBERSORTx suite was used to estimate the immune cell type abundance in bulk RNAseq from MM bone marrow samples from MM patients (N =112) with paired RNA-seq data of purified MM cells as described (21, 22).

## Results

**Figure 1:**
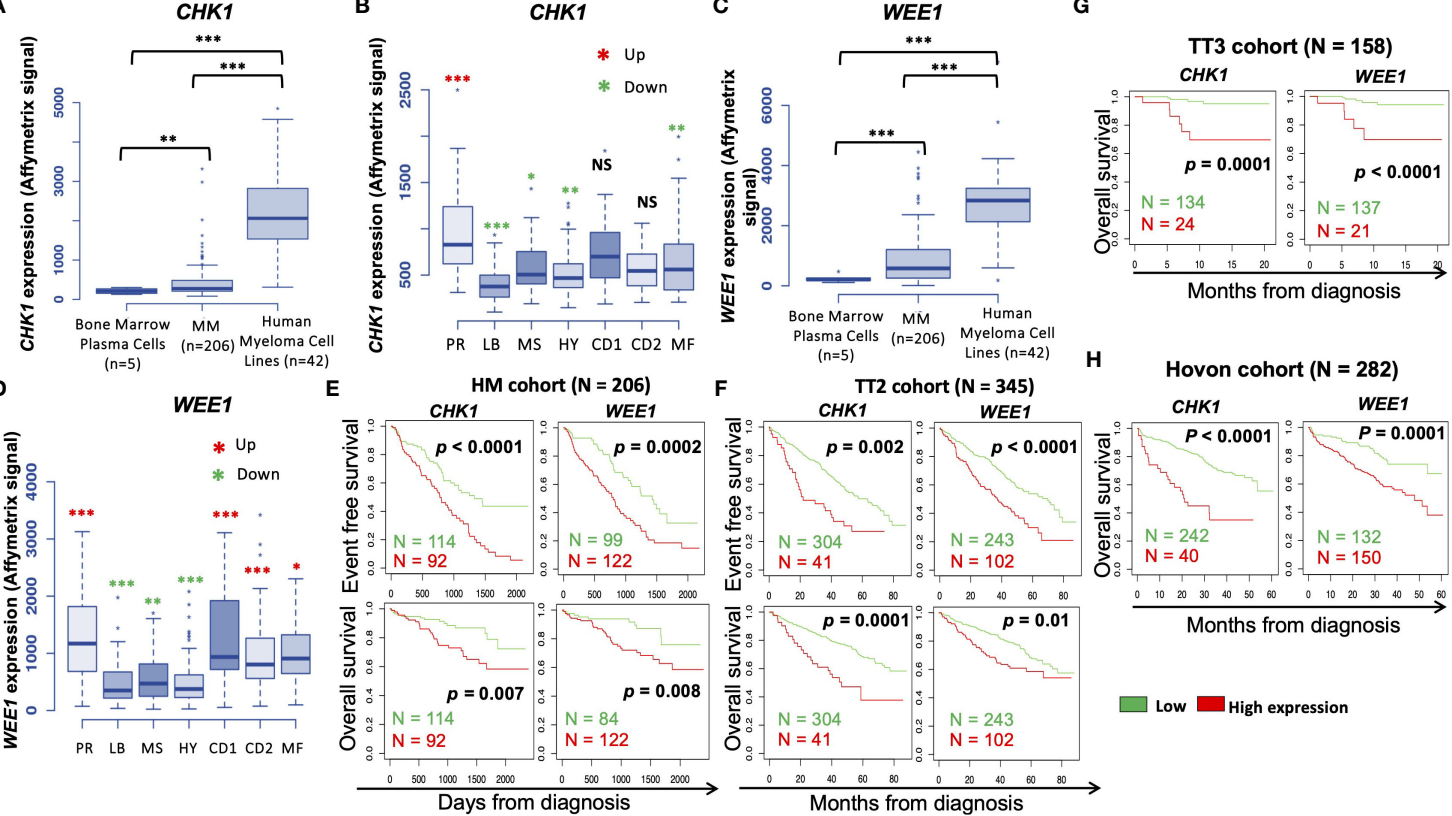
*CHK1* and *WEE1* expression in MM. **(A)** *CHK1* gene expression in normal BMPCs, patients’ MMCs and HMCLs. Data are MAS5-normalized Affymetrix signals (U133 plus 2.0 microarrays). Statistical difference was tested using a Student’s t-test. P-value: **<0.01; ***<0.001. **(B)** Gene expression profiling from MMCs of the patients of UAMS-TT2 cohort were used. Patients were classified in the 7 molecular groups of MM. PR: cell cycle and proliferation, LB: low bone disease, MSET: MMSET overexpression, HY: hyperdiploid signature, CD1: Cyclin D1 and D3 overexpression, CD2: Cyclin D1 and D3 overexpression, MAF: overexpression of c-MAF or MAFB. Red asterisks indicate that *CHK1* expression is significantly higher in the group compared to all the patients of the cohort (P < 0.05) (Student’s t-test). **(C)** *WEE1* gene expression in normal BMPCs, patients’ MMCs and HMCLs. Data are MAS5-normalized Affymetrix signals (U133 plus 2.0 microarrays). Statistical difference was tested using a Student’s t-test. P-value: ***<0.001. **(D)** Gene expression profiling from MMCs of the patients of UAMS-TT2 cohort were used. Patients were classified in the 7 molecular groups of MM. PR: cell cycle and proliferation, LB: low bone disease, MS: MMSET overexpression, HY: hyperdiploid, CD1: Cyclin D1 and D3 overexpression, CD2: Cyclin D1 and D3 overexpression, MAF: overexpression of c-MAF or MAFB. Big red asterisks indicate that *WEE1* expression is significantly higher in the group compared to all the patients of the cohort (P-value: *<0.05; **<0.01; ***<0.001.) (Student’s t-test). High *CHK1* and *WEE1* expression in MMCs could predict for shorter overall and event-free survival (OS and EFS, respectively). Patients of the Heidelberg–Montpellier cohort (N = 206) were ranked according to increasing *CHK1* or *WEE1* expression and a maximum difference in OS and EFS was obtained using the Maxstat R function **(E)**. These results were validated in independent cohorts including the TT2 (n = 345) **(F)**, TT3 (N = 158) **(G)** and Hovon (N = 282) **(H)** cohorts.

**Figure 2:**
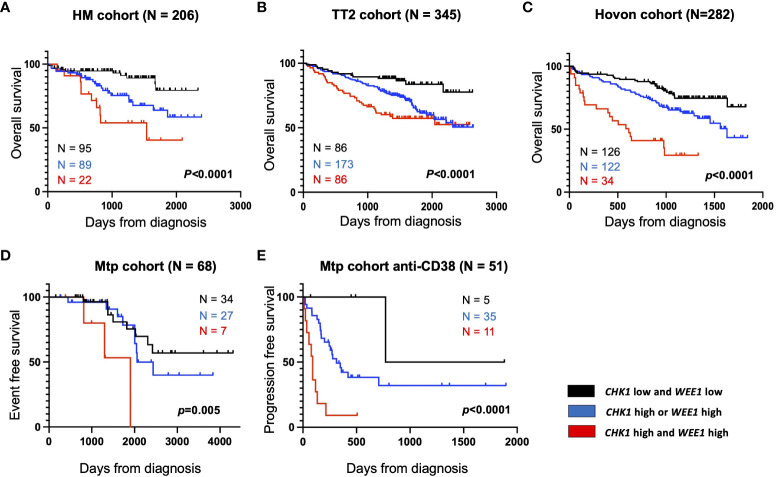
High *CHK1* and *WEE1* expression is associated with a poor outcome in MM. Correlation between *CHK1* and *WEE1* expression and overall survival was analyzed using Maxstat R package in independent cohorts of MM patients (**Supplementary Figure S1**). Combined high expression of *CHK1* and *WEE1* are associated with a poor outcome in 5 independent cohorts including patients at diagnosis treated by HDT (high-dose chemotherapy) and ASCT (autologous stem cell transplant). **(A)** UAMS-TT2 cohort; **(B)** TT3 cohort; **(C)** Hovon cohort; **(D)** Montpellier (Mtp) cohort; a cohort of patients at relapse treated by Anti-CD38 antibody (Daratumumab) **(E)** Mtp cohort anti-CD38 and a cohort of patients at diagnosis non-eligible to HDT and ASCT.

We investigated the prognostic value of *CHK1* and/or *WEE1* gene expression in MM patients. Both *CHK1* and *WEE1* were significantly overexpressed in human myeloma cell lines (HMCLs, n= 42) compared to normal bone marrow plasma cells (BMPCs, n=5) (*p*<0.001 and *p*<0.001 respectively) (**Figures 1A, C**). Furthermore, although we did not observe a statistical difference with normal BMPCs, *CHK1* and *WEE1* expression levels appeared heterogeneous in MMCs ranging from 82 to 3314 and 8 to 4444, respectively (**Figures 1A–D**). *CHK1* and *WEE1* expression were significantly higher in the “proliferation” MM subgroup (23) (*p*<0.001 and *p*<0.001, respectively) (**Figures 1B, D**). Furthermore, *WEE1* expression was also significantly higher in CD1, CD2 and MAF subgroups (23) (**Figures 1B, D**). High *CHK1* expression or high *WEE1* expression alone could predict for shorter overall survival (OS) in 4 independent cohorts of newly-diagnosed patients treated by high dose therapy (HDT) and autologous stem cell transplantation (ASCT) (*p*<0.007 and *p*=0.008 in the HM cohort (N=206; E-MTAB-372), *p*<0.0001 and *p*=0.01 in UAMS-TT2 cohort (N=345; GSE24080), *p*<0.0001 and *p*<0.0001 in Hovon cohort (N=282; GSE19784) and *p*=0.0001 and *p*<0.0001 in UAMS-TT3 cohort (N=186; GSE24080))(**Figures 1E–H**). Same results were obtained for event-free survival (EFS) (**Figures 1E, F**). MM patients from the different cohorts were ranked according to increasing *CHK1* or *WEE1* expression and a maximal difference in OS and EFS was obtained using the Maxstat R function (24). Furthermore, a significant higher expression of *CHK1* and *WEE1* was identified in the patients with del17p compared to patients without del17p in the UAMS TT2 cohort. Furthermore, a higher expression of *CHK1* was identified in patients with 4 or more copies of ch1q. A significant higher expression of *WEE1* was identified in patients with 3 or more copies of ch1q (**Supplementary Figure S1**). Interestingly, concurrent high expression of *CHK1* and *WEE1* is associated with significant poor outcome compared to high expression of *CHK1* or *WEE1* alone in 4 independent cohorts of newly diagnosed patients, including Affymetrix microarrays (**Figures 2A–C**) and RNA-seq data (**Figure 2D**).

**Figure 3:**
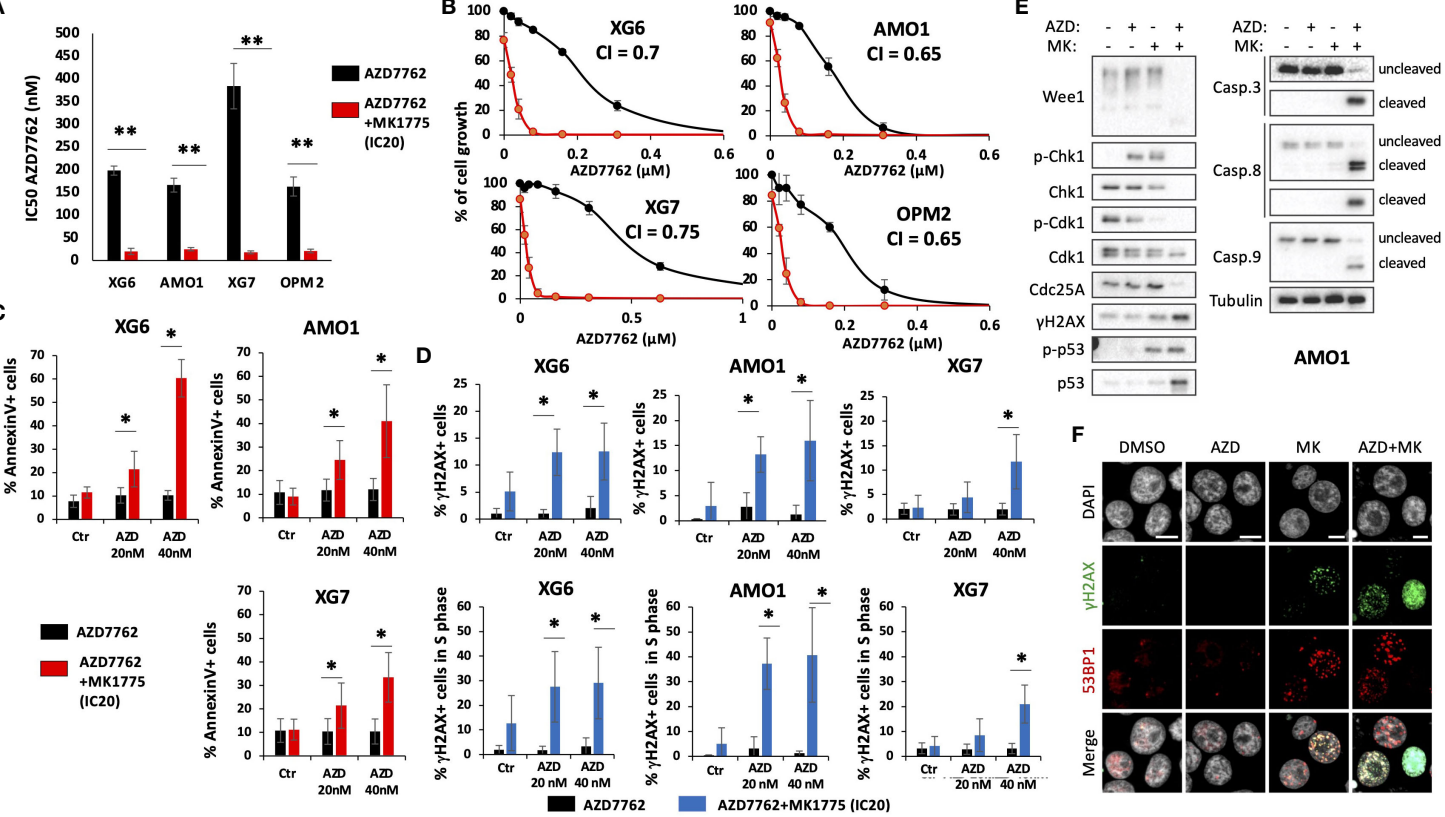
Combination of Chk1 and Wee1 inhibitors synergizes to induce MM cell toxicity. Human myeloma cell lines (HMCLs) were cultured for 4 days in 96-well flat-bottom microtiter plates in RPMI 1640 medium, 10% Fetal calf serum 2ng/ml IL-6 culture medium (control) and increasing AZD7762 concentrations **(A, B)**. IC50 were calculated after viability assessment by CellTiter-Glo luminescent cell viability assay. Results are representative of three independent experiments. Significant synergy and combination index (CI) are calculated by the method of Chou Talalay. P-value: *<0.05; **<0.01. **(C)** IC20 of MK1775 Wee1 inhibitor potentiates AZD7762 Chk1 inhibitor-induced apoptosis (Annexin V) in XG6, XG7 and AMO1 MM cell lines. Annexin V was monitored by flow cytometry after four days of treatment. Results are representative of four independent experiments. Statistical significance was tested using a Student´s t-test for pairs. P-value: *<0.05. **(D)** DNA damage induction was analyzed by measuring γH2AX levels (25) in total and BrdU-positive cells. Results are representative of four independent experiments. Statistical significance was tested using a Student’s t-test for pairs. P-value: *<0.05. **(E, F)** AMO1 cells were treated with AZD7762 (40 nM) and MK1775 (IC20) for 72h as indicated. At the end of the treatments, cells were collected for **(E)** western blot analysis or **(F)** fixed for immunofluorescence with 4% PFA for 10 min at RT to detect γH2AX and 53BP1 foci. DAPI stains DNA. Scale bars = 10 μm. Results are representative of 3 independent experiments.

Furthermore, we also identified that concomitant high *CHK1* and *WEE1* expression is associated with a significant shorter EFS, compared to high *CHK1* or *WEE1* expression alone, in a cohort of patients at relapse treated by anti-CD38 monoclonal antibody (5) (**Figure 2E**). GSEA analysis revealed a significant enrichment of genes involved in metabolism (including cysteine and methionine metabolism, fatty acid metabolism, arginine and proline metabolism), aggrephagy, ATP-binding cassette (ABC) transporters, EGFR signaling and multiple myeloma CD1 and CD2 molecular subgroups (23) (**Supplementary Figure S2**). According to these results, we investigated the therapeutic interest of Chk1 and Wee1 inhibitors (AZD7762 and MK-1775) alone and in combination using our large collection of MM cell lines with different cytogenetic abnormalities (12, 13, 17). Chk1 inhibitor (AZD7762) induced a dose-dependent cell growth inhibition in 13 HMCLs with a median IC50 of 179 nM (range 69–366 nM) (**Supplementary Figure S3A**). A correlation between HMCL sensitivity to Chk1 inhibitor and *TP53* mutation was observed (r=0.7, *p*<0.01, n=13). HMCLs with *TP53* mutation were significantly more sensitive to Chk1 inhibitor treatment (**Supplementary Figure S3B**). Wee1 inhibitor (MK-1775) also induced a dose-dependent inhibition of cell growth in all investigated HMCLs (n=13), with a median IC50 of 450 nM (range 100–800 nM) (**Supplementary Figure S3C**). According to these results, we investigated the therapeutic potential to combine sublethal dose of Wee1 inhibitor (IC20) with increasing doses of Chk1 inhibitor in 4 different HMCLs. A significant synergy (combination index range 0.4-0.7) was identified with a significant reduction of Chk1 inhibitor (AZD7762) IC50 (198 nM to 20 nM for XG6; 166 nM to 25 nM for AMO1; 386 nM to 18 nM for XG7 and 163 nM to 21 nM for OPM2) in all the tested HMCLs (**Figures 3A, B**). According to the significant synergy observed between Chk1 and Wee1 inhibitors, we investigated the effects of the combination on cell cycle, apoptosis and DNA damage induction. Combination of Wee1 inhibitor sublethal concentration (IC20) with different doses of Chk1 inhibitor resulted in significant cell cycle arrest characterized by a decrease of the S-phase population (**Supplementary Figure S4A**), apoptosis potentiation (**Figure 3C**) and PARP1 cleavage (**Supplementary Figure S4B**) in MM cells compared to Chk1 or Wee1 inhibitor alone. The combination of Chk1 and Wee1 inhibitors was also associated with higher DNA double-strand break induction, as evidenced by an increased percentage of γH2AX positive cells in total and proliferating cells (**Figures 3D–F**; **Supplementary Figures S5A–C**). The activation of apoptosis marked by caspases cleavage was confirmed by western blot. These analyses also showed that DNA damage and apoptosis activation correlated with p53 phosphorylation in all cell lines (**Figure 3E**; **Supplementary Figure S5A**).

Wee1 and Cdk1 kinases and Cdc25 phosphatases act in a feedback loop to control the timely entry into mitosis (26, 27). In G2, Wee1 inhibits Cdk1 by phosphorylation on Tyr15. During G2/M transition, Cdc25s dephosphorylate Cdk1, allowing an increase of Cdk1 activity that will trigger Wee1 degradation (28–30), further boosting the Cdk1 activation and promoting the entry into mitosis (27, 31). As expected, MK1775 treatment led to dephosphorylation of Tyr15-Cdk1, that was stronger in combination with AZD7762. Cdk1 activation correlated with a significant degradation of Wee1 in AMO1 and XG6 cell lines. However, no clear effect was observed on Cdc25A (**Figure 3E**; **Supplementary Figure S5A**). We therefore conclude that combined targeting of Chk1 and Wee1 demonstrates synergistic toxicities in MM cells characterized by DNA damage, induction of cell cycle dysregulation and triggering of caspases-dependent apoptosis.

**Figure 4:**
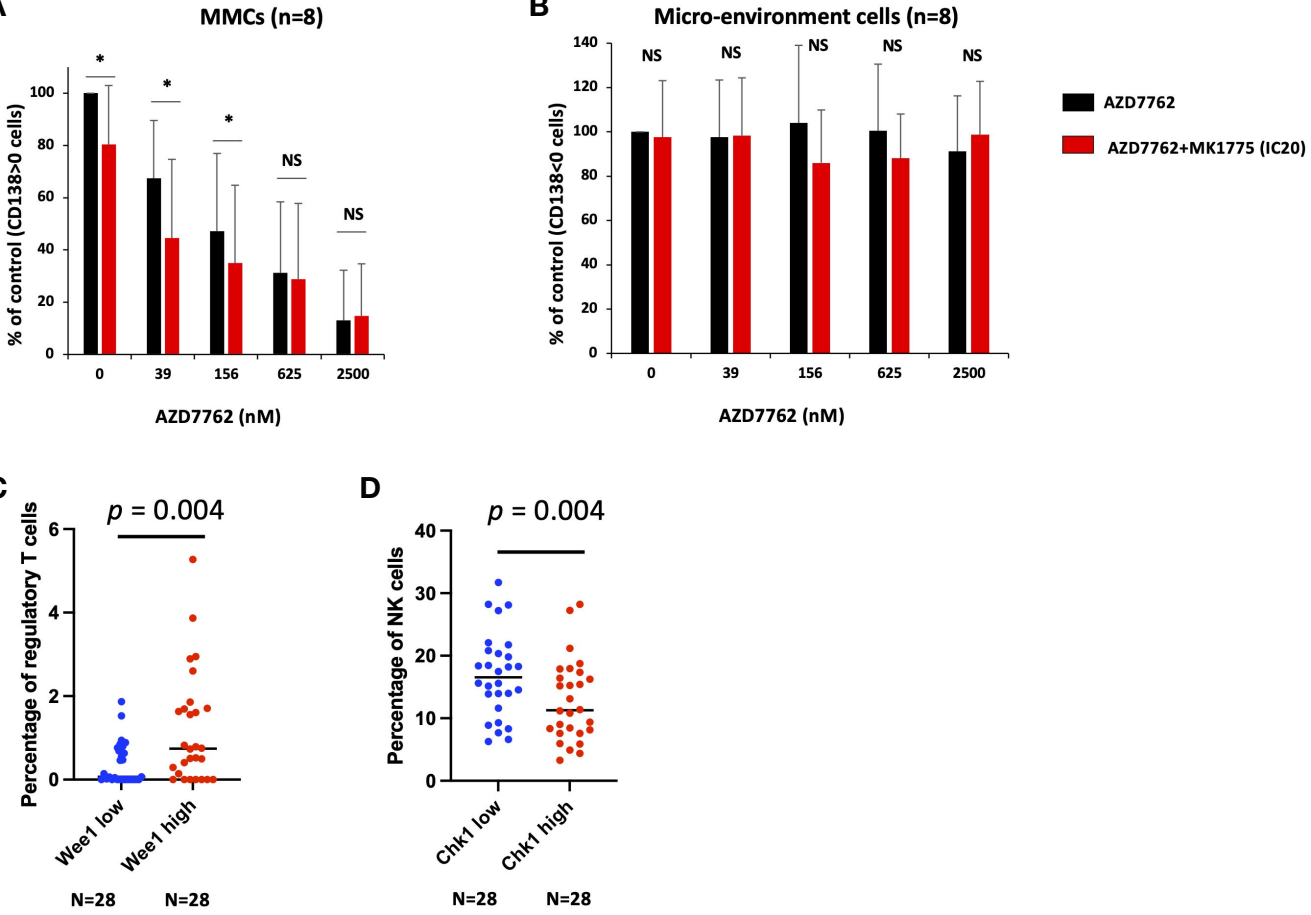
Chk1 and Wee1 inhibitors synergize to induce primary MM cell death. Mononuclear cells from eight MM patients were treated or not with graded concentrations of Chk1 inhibitor and IC20 of Wee1 inhibitor. At day 4 of the culture, the viability and total cell counts were assessed and the percentage of CD138^+^ viable plasma cells **(A)** and bone marrow non-myeloma cells **(B)** were determined by flow cytometry. Results are median values of the percentage of myeloma and micro-environment cells in the culture wells. Results were compared with a Student´s t-test for pairs. P-value: *<0.05; NS: not significant. **(C, D)** Using CIBERSORTx suite, we estimated the immune cell type abundance in bulk RNAseq from MM bone marrow samples (N = 112). We compared the patients with high (Q4, n=28) and low (Q1, n=28) *WEE1* or *CHK1* expression and the percentage of the different immune cell populations. Results were compared with a Student´s t-test.

Of interest, we validated the therapeutic interest of Chk1i/Wee1 inhibitor combination on primary MM cells of patients co-cultured with their bone marrow microenvironment (**Figure 4A**). This toxicity is specific of MM cells since normal bone marrow cells were not significantly affected by the combination treatment (**Figure 4B**). Taken together, our data suggest that the association of Chk1 or Wee1 inhibitors may represent a promising therapeutic approach in high-risk MM patients characterized by high *CHK1* and *WEE1* expression. The poor outcome associated with high *CHK1* and *WEE1* expression may be associated with a significant advantage to cope with DNA insults generated by DNA-damaging agents used in MM. We previously reported that a low dose of Chk1 inhibitor significantly potentiates melphalan toxicity in MM cells (25). We confirmed these results using additional MM cell lines and also demonstrated that IC20 of Wee1 inhibitor significantly potentiates melphalan toxicity in three HMCLs (**Supplementary Figures S6A, B**). This data underlined the potential role of Chk1 and Wee1 in melphalan resistance in association with the observed poor outcome in cohorts of patients treated by HDT and ASCT.

Of interest, we identified that *CHK1* and *WEE1* expression are also associated with a poor outcome in a cohort of patients at relapse treated by anti-CD38 MoAb. Using a cohort of 112 MM patients including the RNAseq data of purified MM cells together with RNA-seq data of the non-tumor bone marrow fraction, we investigated the correlation between CHK1 and WEE1 expression in MM cells and the abundance of immune cell subpopulations within the paired bone marrow samples. Using the CIBERSORTx suite (21), we estimated the immune cell type abundance in bulk RNA-seq from MM bone marrow samples. Of interest, we identified a significant correlation between *WEE1* expression by the plasma cells and the percentage of regulatory cells in the bone marrow (r = 0.28; p < 0.05; n=112). Furthermore, a comparison of patients with high (Q4, n=28) and low *WEE1* expression (Q1, n=28) revealed a significant 3-fold increase in regulatory T cells in the *WEE1* ^high^ subgroup (*p* = 0.004) (**Figure 4C**). A significant inverse correlation was also identified between *CHK1* expression in MM cells and the percentage of NK cells within the bone marrow (r = -0.20; p < 0.05; n=112). Comparing patients with high (Q4, n=28) and low *CHK1* expression (Q1, n=28) underlined a significant decrease in the percentage of NK cells in the *CHK1* ^high^ subgroup (*p* = 0.02) (**Figure 4D**). DNA damage response deficiency and replication stress have recently emerged as an important determinant of tumor immunogenicity. The overexpression of Wee1 and Chk1 may contribute to MM cell adaptation to replicative stress and may decrease the activation of the cell-intrinsic innate immune response and the response to immunotherapy.

Altogether, these data suggest that the association of Chk1 and Wee1 inhibitors may represent a promising therapeutic approach in high-risk MM patients characterized by high *CHK1* and *WEE1* expression.

## Discussion

In this manuscript, we identified that concomitant overexpression of *CHK1* and *WEE1* is associated with a poor outcome in MM including newly diagnosed MM patients treated by high dose melphalan and ASCT and patients at relapse treated with anti-CD38 MoAb. The combination of Chk1 and Wee1 inhibitors demonstrates synergistic toxicities in MM cells in association with DNA damage and apoptosis induction without significant toxicity on normal bone marrow cells. In MM, a subgroup of patients is characterized by replicative stress, chromosomal instability, and a poor outcome (32). Furthermore, MYC deregulation generate replicative stress and DNA damage response in MM cells (32). Our group previously reported that overexpression of RECQ DNA helicases is associated with adaptation to replication stress and resistance to conventional therapeutic agents in MM (5, 6). Furthermore, we identified *CHK1* as a poor prognostic factor in MM among others kinase related-genes (25). Wee1 plays a critical role in the activation of the G2-M checkpoint but is not directly regulated by DNA damage unlike Chk1 (33). Clinical development has concentrated on using Chk1 inhibitors in combination with drugs that cause replication-dependent DNA damage since preclinical research has shown that these approaches have the greatest synergy (34). Wee1 inhibition prolongs mitosis and makes cancer cells more susceptible to chemotherapy-induced mitotic catastrophe together with apoptosis induction. Furthermore, Wee1 inhibition was shown to synergize with bortezomib in MM (9). Interestingly, we identified that patients with high *CHK1/WEE1* co-expression are associated with a significant enrichment in genes involved in metabolism, ABC transporters, aggrephagy, EGFR signaling pathway and upregulated in CD1 and CD2 MM molecular subgroups. EGF family members have been described as MM cell growth factors (35, 36). Overexpression of ABC transporters is an important mechanism of multidrug resistance in cancer (3, 37). The energy produced by ATP hydrolysis is used by this class of transmembrane proteins to export cytotoxic compounds.

MM cells are characterized by sustained endoplasmic reticulum (ER) stress and unfolded protein response (UPR) activation due to high immunoglobulin secretion. Aggrephagy is a type of autophagy playing an important role to degrade misfolded proteins (38). Plasma cells with impaired autophagy display increased immunoglobulin production, decreased intracellular ATP and elevated ER stress (39). Targeting protein homeostasis in MM is widely used with proteasome inhibitors to exploit the elevated misfolded protein accumulation in plasma cells (40). Metabolism deregulation is also involved in MM pathophysiology (41) and drug resistance (42, 43).

We found that combination of low doses of Chk1 and Wee1 inhibitors synergize to induce major toxicity in MM cells with a significant increase of spontaneous DNA damage and apoptosis induction. Of note, the drug combination induced also a significant death of patients’ MM cells without affecting the survival of non-tumor bone marrow environment cells. Our data demonstrate that targeting Chk1 and Wee1 could have a therapeutic potential in Chk1^high^/Wee1^high^ MM patients associated with a poor outcome. In spite of effective therapeutic protocols developed in MM, drug resistance remains a major concern. High *CHK1* and *WEE1* expression may be related with a considerable benefit in MM patients’ ability to deal with DNA insults caused by genotoxic agents. We stated that a low dose of Chk1 inhibitor dramatically increases the toxicity of melphalan in MM cells (25) and showed that low dose of Wee1 inhibitor greatly increases the melphalan toxicity in HMCLs. Of note, these drugs could be associated with significant toxicities. Synthetic lethality between different DNA repair inhibitors or with DNA-damaging drugs might enable the latter to be utilized at lower concentrations, limit mutagenic effects, and reduce unfavorable side effects.

An exciting emerging concept is the link between replication stress and activation of the cell-intrinsic innate immune response (44). Combination approaches integrating replication stress–inducing agents, such as carboplatin or gemcitabine, with immunotherapies like the immune checkpoint inhibitor nivolumab, have advanced to clinical trials (45). The immunogenic properties of conventional therapeutics such as genotoxic agents (46, 47) or Bortezomib (48) have revealed their therapeutic interest to enhance anti-neoplastic immune responses in cancer including MM. A growing body of evidence now supports the concept that DDR-targeted therapies can increase the antitumor immune response by promoting antigenicity through increased mutability and genomic instability and enhancing immunogenic cell death through the modulation of factors that control the tumor–immune cell synapse (44, 49). In association with the poor outcome of MM patients with high *CHK1/WEE1* expression following anti-CD38 MoAb treatment, we identified that patients with high *CHK1* expression exhibit a significant lower percentage of NK cells in the bone marrow whereas patients with high *WEE1* expression display a significant higher percentage of regulatory T cells. These data suggest that MM cell adaptation to replicative stress through Wee1 and Chk1 upregulation may decrease the activation of the cell-intrinsic innate immune response and the response to immunotherapy. Of interest, a recent study reported that Chk1 inhibitor treatment promotes proinflammatory cytokine expression, innate cell immune response and tumor regression in melanoma models (50). Yet, the toxicity of Chk1 and Wee1 inhibitors’ association need to be carefully addressed using *in vivo* models to assess the benefits and risks of this combination in MM.

## Data availability statement

The datasets presented in this study can be found in online repositories. The names of the repository/repositories and accession number(s) can be found in the article/**Supplementary Material**.

## Ethics statement

Ethical approval was not required for the studies on humans in accordance with the local legislation and institutional requirements because only commercially available established cell lines were used.

## Author contributions

AB: Formal Analysis, Investigation, Methodology, Writing – original draft. LD: Formal Analysis, Investigation, Methodology, Writing – original draft. HdB: Formal Analysis, Investigation, Methodology, Writing – original draft. TM: Formal Analysis, Investigation, Methodology, Writing – original draft. DC: Formal Analysis, Investigation, Methodology, Writing – original draft. NR: Formal Analysis, Investigation, Methodology, Writing – original draft. GR: Formal Analysis, Investigation, Methodology, Writing – original draft. GC: Data curation, Investigation, Writing – original draft. LV: Data curation, Investigation, Writing – original draft. CH: Conceptualization, Data curation, Investigation, Writing – original draft. ML: Data curation, Investigation, Methodology, Writing – original draft. CB: Conceptualization, Data curation, Investigation, Methodology, Validation, Writing – original draft. PP: Conceptualization, Investigation, Methodology, Validation, Writing – original draft. JM: Conceptualization, Funding acquisition, Methodology, Project administration, Supervision, Validation, Writing – original draft, Writing – review & editing. SO: Conceptualization, Investigation, Methodology, Project administration, Supervision, Validation, Writing – original draft, Writing – review & editing.
