# Supplementary material for: Combined inhibition of Wee1 and Chk1 as a therapeutic strategy in multiple myeloma
Source: Front Oncol. 2023 Dec 6;13:1271847. doi: 10.3389/fonc.2023.1271847 (PMC10730928; doi:10.3389/fonc.2023.1271847)
Supplement: Supplementary file 1 [file DataSheet_1.pdf]

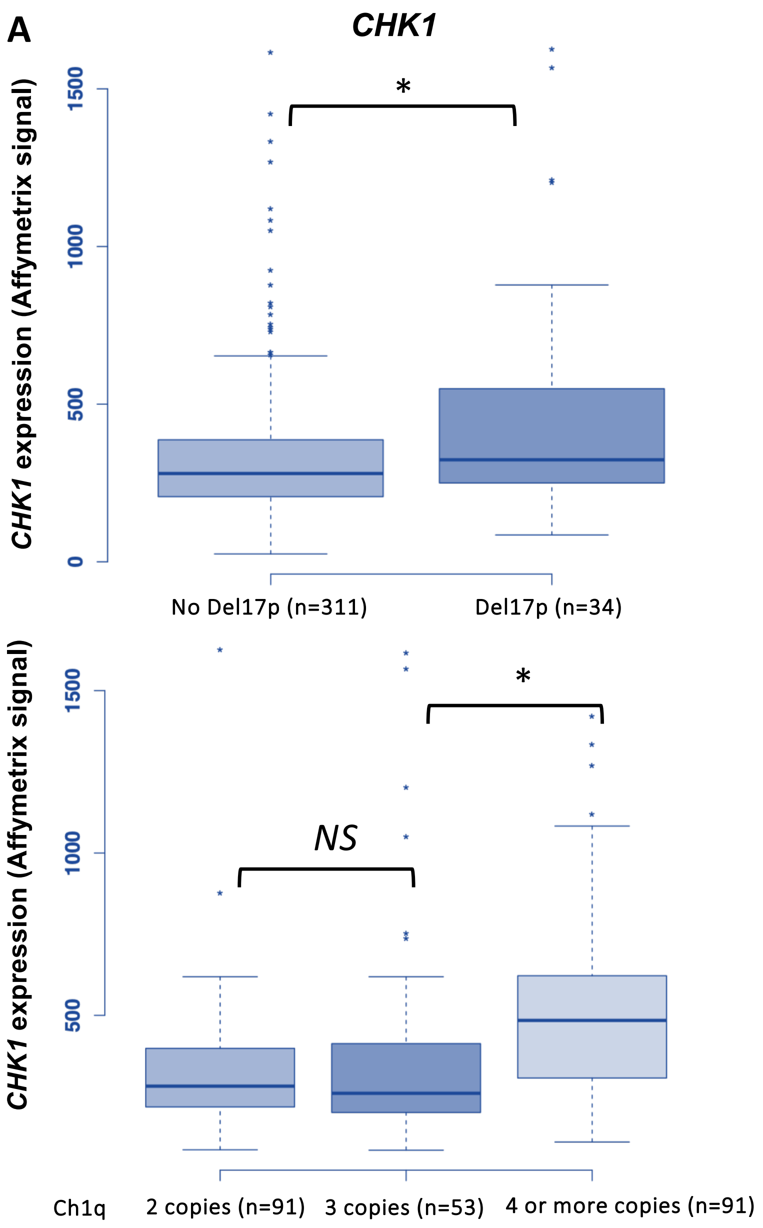
A
CHK1
CHK1 expression (Affymetrix signal)
0
500
1000
1500
*
No Del17p (n=311)
Del17p (n=34)
B
WEE1
WEE1 expression (Affymetrix signal)
0
1000
2000
3000
4000
5000
6000
*
No Del17p (n=311)
Del17p (n=34)
CHK1 expression (Affymetrix signal)
500
1000
1500
NS
*
Ch1q
2 copies (n=91)
3 copies (n=53)
4 or more copies (n=91)
WEE1 expression (Affymetrix signal)
0
1000
2000
3000
4000
5000
6000
*
Ch1q
2 copies (n=91)
3 copies (n=53)
4 or more copies (n=91)

Supplementary Figure S1

CHK1 and *WEE1* expression was investigated in patients of the UAMS TT2 cohort comparing (A) patients with or without del17p or (B) with or without ch1q gain. Data are MAS5-normalized Affymetrix signals (U133 plus 2.0 microarrays). Statistical difference was tested using a Student t-test: * p-value < 0.05.

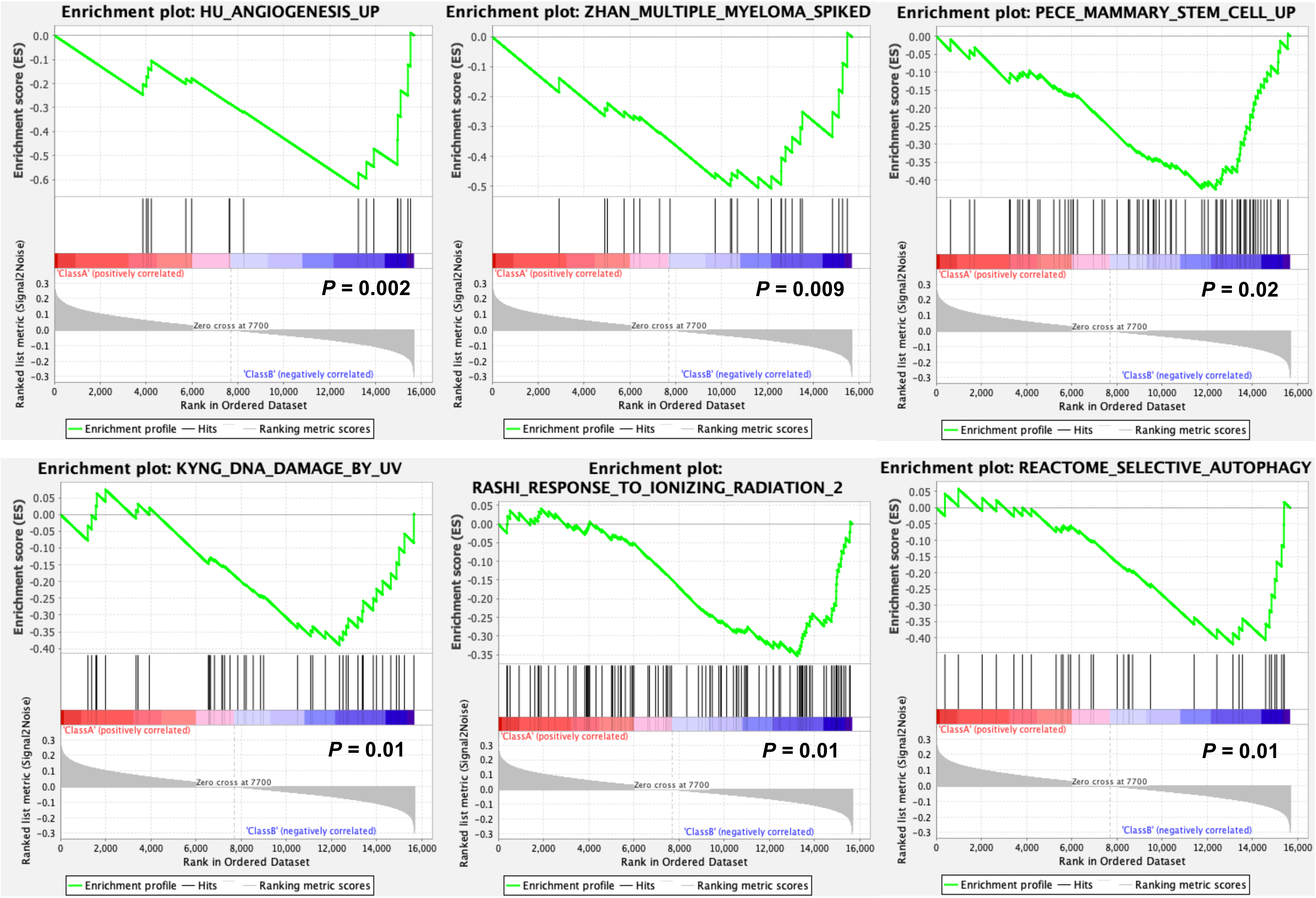

Enrichment plot: HU_ANGIOGENESIS_UP
P = 0.002
Enrichment plot: ZHAN_MULTIPLE_MYELOMA_SPIKED
P = 0.009
Enrichment plot: PECE_MAMMARY_STEM_CELL_UP
P = 0.02
Enrichment plot: KYNG_DNA_DAMAGE_BY_UV
P = 0.01
Enrichment plot: RASHI_RESPONSE_TO_IONIZING_RADIATION_2
P = 0.01
Enrichment plot: REACTOME_SELECTIVE_AUTOPHAGY
P = 0.01
Enrichment score (ES)
Ranked list metric (Signal2Noise)
Rank in Ordered Dataset
'ClassA' (positively correlated)
'ClassB' (negatively correlated)
Zero cross at 7700
Enrichment profile
Hits
Ranking metric scores

Supplementary Figure S2

Top gene set significantly associated with concomitant high *CHK1* and *WEE1* expression in MM**.** Gene Set Enrichment Analysis (GSEA) enrichment plots with the absolute enrichment p-value were completed using the UAMS TT2 cohort.

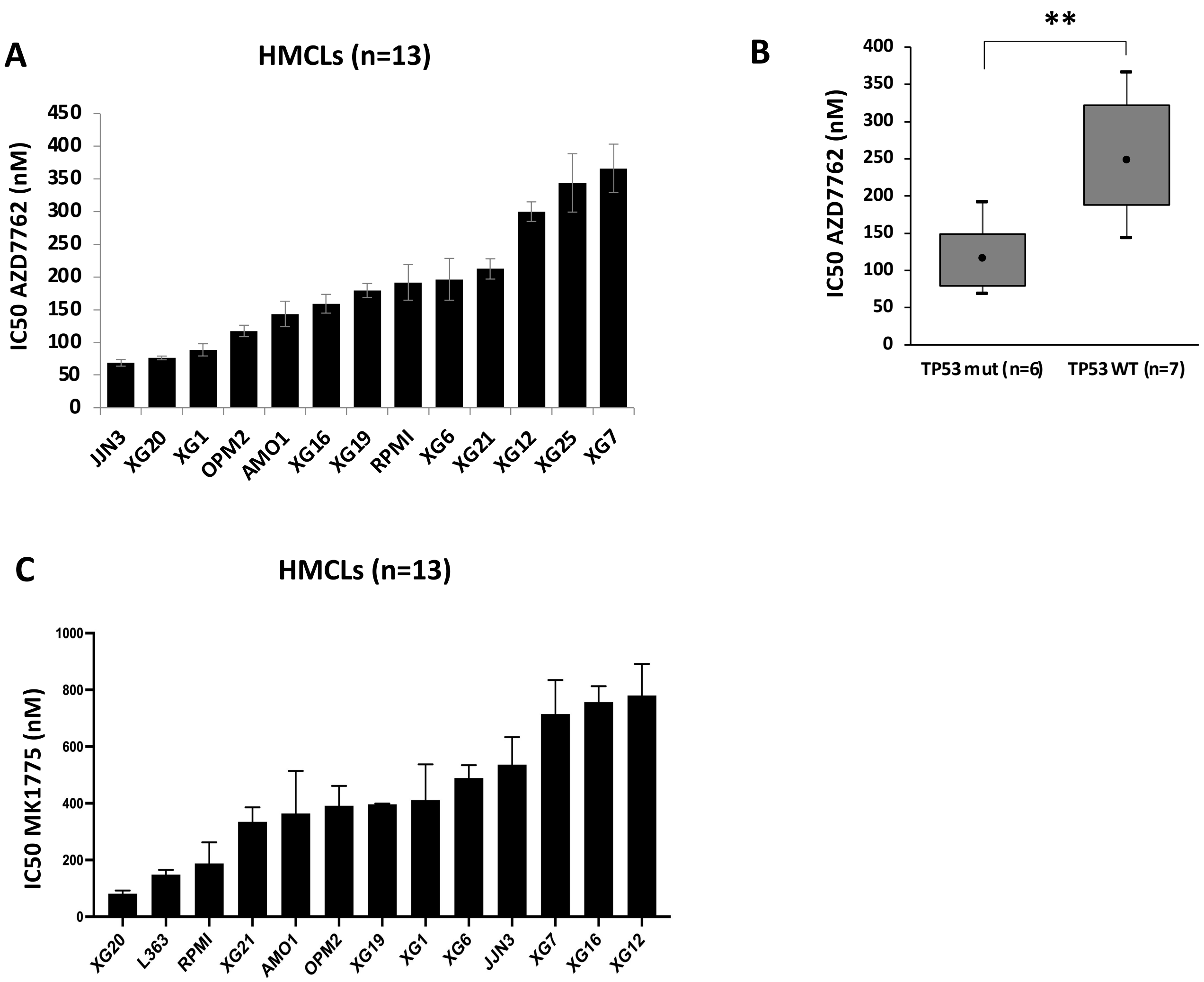

Supplementary Figure S3

Supplementary Figure S3

Effect of AZD7762 and MK1775 in HMCLs. **(A)** 13 HMCLs were treated with increasing doses of AZD7762. At day 4, cell viability was assessed using CellTiter-Glo Luminiscent Cell Viability Assay. IC50 for each cell line was calculated using GraphPrism software. Data are based on at least 3 independent experiments. **(B)** HMCLs with *TP53* mutation are significantly more sensitive to AZD7762 treatment than cell lines with *TP53* wild type according to their IC50. **(C)** 13 HMCLs were treated with increasing doses of MK1775. At day 4, cell viability was assessed using CellTiter-Glo Luminiscent Cell Viability Assay. IC50 for each cell line was calculated using GraphPrism software. Data are based on at least 3 independent experiments.

Supplementary Figure S4

A

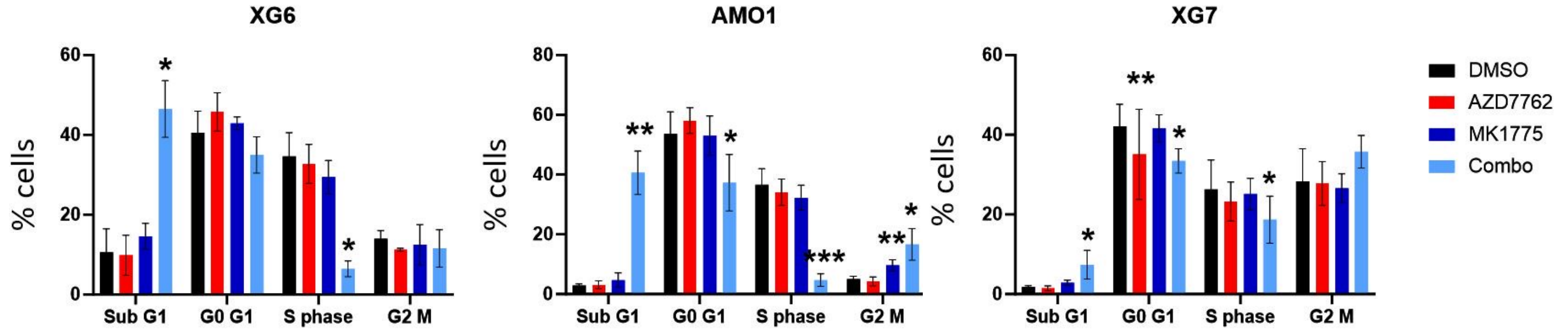

B

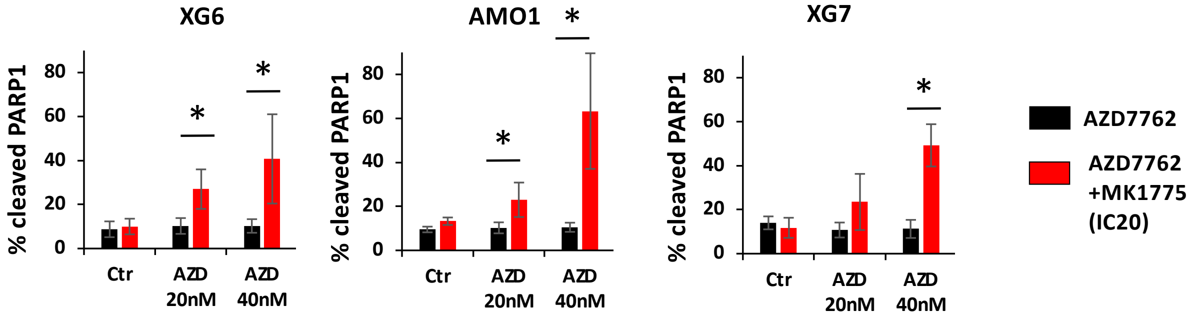

Supplementary Figure S4

(A) Cells were treated 72h with AZD7762 (40nM) and MK1775 (IC20) as indicated. BrdU (10 µg/ml) was added during the last 1.5 hours of treatment. Cells were fixed and processed to detect BrdU incorporation and total DNA. BrdU+ cells were assigned to S-phase. BrdU- cells were assigned to G0/G1 or G2/M phases based on their DNA content. * indicates a significant difference compared to DMSO treated cells after applying a Student's t-test for pairs. Results are the mean of 3 independent experiments. **(B)** Combination of AZD7762 and MK1775 inhibitors induces MM cell apoptosis (PARP cleavage) in XG6, XG7 and AMO1 MM cell lines. PARP cleavage was monitored by flow cytometry after four days of treatment. Results are the mean of four independent experiments. Statistical significance was tested using a Student t-test for pairs. P-value: *<0.05; **<0.01.

Supplementary Figure S5

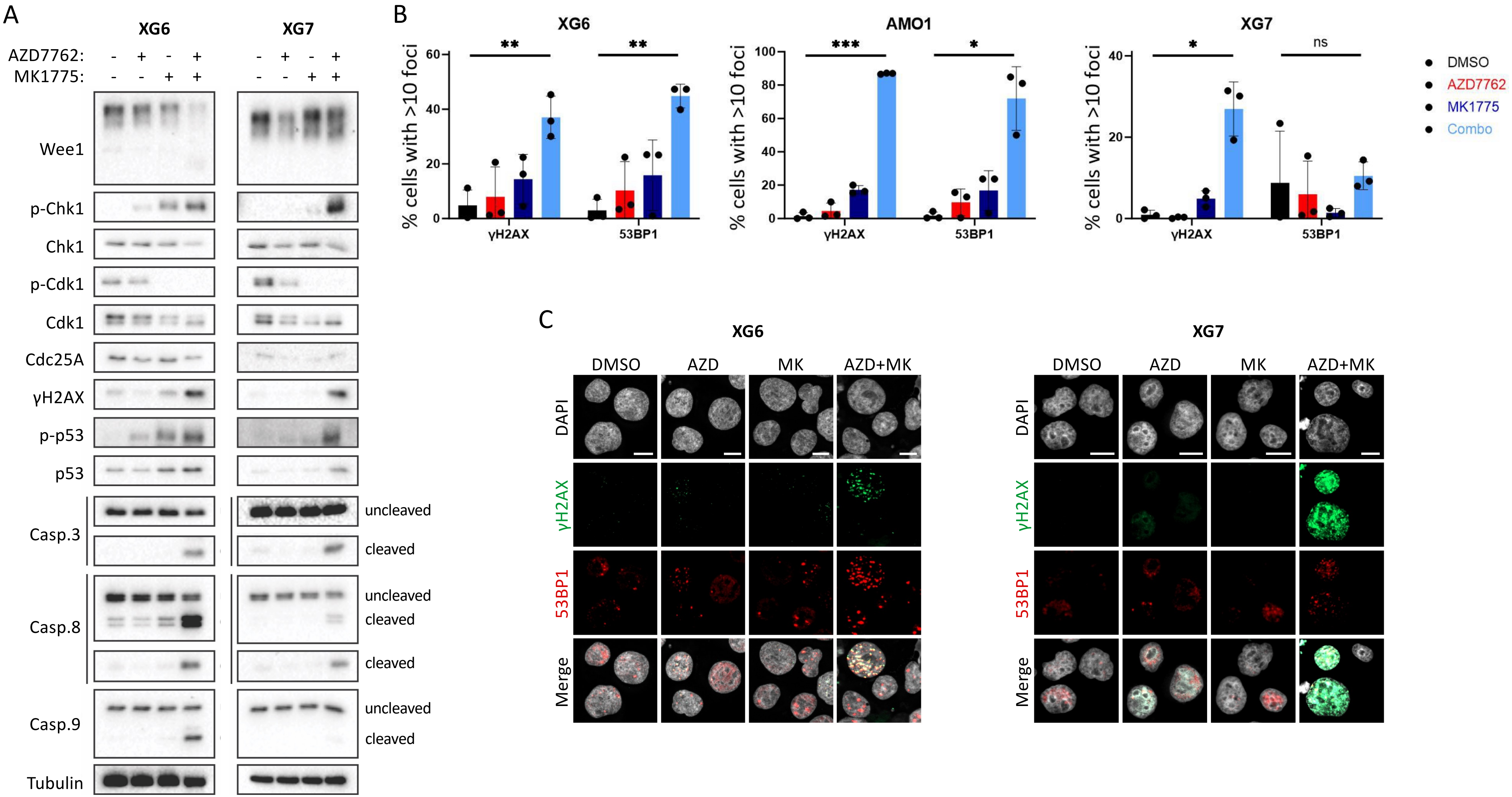

Supplementary Figure S5. Cells were treated with AZD7762 (40nM) and MK1775 (IC20) for 72h as indicated. **(A)** At the end of the treatments, cells were collected for western blot analysis and **(B,C)** fixed with 4% PFA for 10 min at RT. Immunofluorescence with specific antibodies was performed to detect γH2AX and 53BP1 nuclear foci. The graphs in (B) show the quantification of the percentage of cells in the population with more than 10 γH2AX foci. A minimum of 200 cells were analyzed per condition. Results show the mean and standard deviation of 3-4 independent experiments. Student's t-test: * p-value < 0.05, ** p-value < 0.001, *** p-value < 0.005. (C) Representative images of cells quantified in (B). Scale bars = 10 μm.

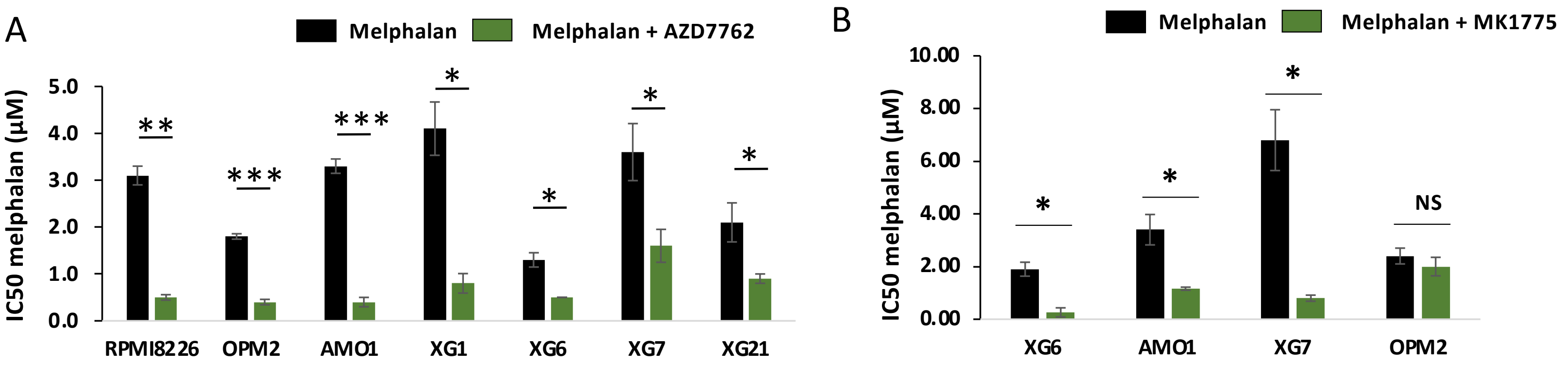

Supplementary Figure S6

Supplementary Figure S6

Chk1 and Wee1 inhibitors enhance the sensitivity of MM cells to melphalan. Human myeloma cell lines (HMCLs) were cultured for 4 days in 96-well flat-bottom microtiter plates in RPMI 1640 medium, 10% Fetal calf serum 2ng/ml IL-6 culture medium (control) and graded melphalan concentrations in presence or absence of IC20 of (A) or (B) MK1775. IC50 were calculated after viability assessment by CellTiter-Glo luminescent cell viability assay. Results are representative of four independent experiments. Statistical significance was tested using a Student t-test for pairs. P-value: *<0.05; **<0.01. NS: not significant.
